# Farnesyltransferase-inhibitors exert *in vitro* immunosuppressive capacity by inhibiting human B-cells

**DOI:** 10.3389/frtra.2023.1233322

**Published:** 2023-11-09

**Authors:** Shilei Xu, Sebastian Dolff, Nils Mülling, Hagen S. Bachmann, Yang Dai, Monika Lindemann, Ming Sun, Oliver Witzke, Andreas Kribben, Benjamin Wilde

**Affiliations:** ^1^ Department of Nephrology, University Hospital Essen, University of Duisburg-Essen, Essen, Germany; ^2^ Department of General Surgery, The Third Affiliated Hospital, Sun Yat-Sen University, Guangzhou, China; ^3^ Department of Infectious Diseases, University Hospital Essen, University of Duisburg-Essen, Essen, Germany; ^4^ Institute of Pharmacology and Toxicology, Centre for Biomedical Education and Research, Witten/Herdecke University, Witten, Germany; ^5^ Institute for Transfusion Medicine, University Hospital Essen, University of Duisburg-Essen, Essen, Germany

**Keywords:** B-cells, plasma cells, farnesyltransferase inhibitors, renal transplantation, humoral rejection

## Abstract

**Objectives:**

Farnesyltransferase inhibitors (FTI), which inhibit the prenylation of Ras GTPases, were developed as anti-cancer drugs. As additional target proteins for prenylation were identified in the past, it is likely that FTI have potential value for therapeutic purposes beyond cancer. The effect of FTI on B-cells remains unclear. To address this issue, we investigated the effects of *in vitro* FTI treatment on effector and regulatory B-cells in healthy controls and renal transplant patients.

**Methods:**

For this purpose, B-cells were isolated from the peripheral blood of healthy controls and renal transplant patients. Purified B-cells were stimulated via Toll-like-receptor 9 (TLR-9) in the presence or absence of FTI. Regulatory functions, such as IL-10 and Granzyme B (GrB) secretion, were assessed by flow cytometry. In addition, effector B-cell functions, such as plasma cell formation and IgG secretion, were studied.

**Results:**

The two FTI Lonafarnib and tipifarnib both suppressed TLR-9-induced B-cell proliferation. Maturation of IL-10 producing B-cells was suppressed by FTI at high concentrations as well as induction of GrB-secreting B-cells. Plasma blast formation and IgG secretion were potently suppressed by FTI. Moreover, purified B-cells from immunosuppressed renal transplant patients were also susceptible to FTI-induced suppression of effector functions, evidenced by diminished IgG secretion.

**Conclusion:**

FTI suppress *in vitro* B-cell proliferation and plasma cell formation while partially preserving IL-10 as well as GrB production of B-cells. Thus, FTI may have immunosuppressive capacity encouraging further studies to investigate the potential immunomodulatory value of this agent.

## Introduction

Antibody-mediated rejection (AMR) is widely recognized as the leading cause of late transplant failure and accounts for renal allograft losses. Current therapeutic strategies for treating AMR primarily focus on B-cells, including the use of plasmapheresis, immunoadsorption, and rituximab (1, 2). However, extensive adverse side effects of these approaches severely limit the application (1, 2). The lack of effective treatment to prevent the development of antibody-mediated rejection deepens the need for clinicians to focus on primary prevention of *de novo* humoral allosensitization. Farnesyltransferase inhibitors (FTI), which inhibit the activity of the enzyme Farnesyltransferase, have been investigated extensively because of their multiple biological activities (3–6). Studies have indicated the anti-inflammatory and immunosuppressive functions of FTI (4). FTI inhibit the proliferation of T lymphocytes and prevent graft-vs.-host disease in mice by suppressing expansion of alloreactive T-cells (7, 8). In addition, FTI decrease synovial TNF and IL-1 mRNA expression in rheumatoid arthritis (RA) (9). Moreover, FTI not only reduce neutrophil recruitment but also attenuate acute lung injury provoked by the streptococcal M1 protein (10). FTI are used as experimental treatment in allograft rejection such as acute rejection (11). To date, few studies have explored the effects of FTI on B-cells. In animal studies, FTI blocked proliferation of BCR-expressing B-cell lymphomas by interfering with the antigen receptor and/or cytokine signaling pathways within transformed cells (12). Thus, FTI might have a suppressive effect on B-cell activation. Whereas the effects of FTI on T cells have been studied by several groups, less is known about the impact of FTI on B-cell effector and regulatory function. The aim of this study was to assess the *in vitro* effect of FTI on B-cells in healthy controls and renal transplant patients.

## Materials and methods

### Patients and samples

Buffy coats from healthy blood donors serving as healthy controls (HC) were provided by the Institute for Transfusion Medicine and were used for the experiments. In addition, 26 patients were enrolled after renal transplantation. Details on the patient demographics are given in Table 1. This study was approved by the local ethics committee, and all patients provided informed consent.

**Table 1:** Patient characteristics.

	Renal transplant patients (*n* = 26)
Age, mean ± SD, years	53 ± 10
Sex (female/male)	10/16
Time since transplantation (months)	102 ± 96
# of patients ≥1 previous RTX	6
# of HLA-mismatches (HLA-A,-B,-DR)	3 ± 2
# of patients with DSA	9
Immunosuppressants at the time of sampling (# of patients treated)	
Tacrolimus	23
Cyclosporine A	2
Mycophenolate	15
mTOR inhibitor	6
Leflunomide	1
Steroids	23
CMV status at the time of RTX (D/R)	
+/+	8
+/-	2
-/+	5
-/-	8
Unknown	3

### Peripheral blood mononuclear cell isolation

Peripheral blood mononuclear cells (PBMCs) were isolated by density gradient centrifugation using Lymphoprep (Stemcell, Cologne, Germany). B-cells were isolated from PBMCs using a bead-/column-based magnetic separation method (Miltenyi Biotec, Bergisch Gladbach, Germany). B-cells were purified by negative selection, and purity was typically above 90%. When B-cells were isolated from renal transplant patients, a direct isolation method from whole blood was used. This method is a magnetic bead-based negative selection system (MACSExpress, Miltenyi Biotec), and purity was typically above 90%. To track proliferation, cells were labeled with 2 µM carboxyfluorescein-succinimidyl-ester (CFSE, Sigma–Aldrich, Taufkirchen, Germany).

### Cell culture

B-cell cultures were performed as described previously (13). Isolated B-cells were cultured in RPMI1640 GlutaMAX (Thermo Fisher Scientific, Darmstadt, Germany) at a concentration of 0.5 × 10^6^ cells/ml supplemented with 100 U/ml penicillin (Sigma–Aldrich), 100 µg/ml streptomycin (Sigma–Aldrich), 10% fetal calf serum (Greiner-Bio one, Frickenhausen, Germany), nonessential amino acids (NEAA; Sigma–Aldrich) and sodium pyruvate (Sigma–Aldrich) in 96-well U-bottom plates (Sigma–Aldrich). For the plasmablast formation assay and the phenotyping of costimulatory molecules on B-cells, PBMC were cultured. Depending on the assay, the cells were cultured for three to six days in a 5% CO_2_ atmosphere at 37°C. To induce the production of IL-10**^pos^** B-cells, isolated B-cells were stimulated with CpG (ODN2006, 0.1 *μ*M, InvivoGen, Toulouse, France) for 72 h, followed by restimulation with phorbol 12-myristate 13-acetate (PMA) (10 ng/ml) and ionomycin (1 µg/ml, both Sigma–Aldrich) in the presence of brefeldin A (5 µg/ml, BFA, BD Biosciences). For detection of Granzyme B (GrB**^pos^**) B-cells, isolated B-cells were stimulated with CpG (ODN2006, 0.1 *μ*M) in the presence of IL-2 (50 ng/ml, Miltenyi Biotec) or IL-21 (50 ng/ml, Miltenyi Biotech) for 72 h, followed by restimulation with phorbol 12-myristate 13-acetate (PMA, 10 ng/ml, Sigma–Aldrich) and ionomycin (1 µg/ml, Sigma–Aldrich) in the presence of brefeldin A (BFA, BD Biosciences). For the plasmablast formation assay, unseparated B-cells were stimulated for six days with either CpG (ODN2006, 0.1 *μ*M, Invitrogen) in the presence of IL-2 (50 ng/ml, Miltenyi Biotech) or IL-21 (50 ng/ml, Miltenyi Biotech). To detect IgG secretion, isolated B-cells were stimulated with Poly-S (resiquimod plus IL-2, 1:1000 final dilution, CTL Europe GmbH) for four days and then transferred to a prepared ELISpot plate for incubation for an additional 24 h. The two FTI lonafarnib (Selleckchem, TX, USA) and tipifarnib (Selleckchem, TX, USA) were used at different concentrations for the assays. Tacrolimus (1.25 ng/ml, Sigma–Aldrich) and rapamycin (12.5 ng/ml, Sigma–Aldrich) were used in selected experiments.

### ELISpot assay

For the detection of IgG-secreting human cells, a commercially available ELISpot kit was used (Human IgG Single-Color ELISpot, CTL Europe GmbH) according to the manufacturer's instructions. For the final incubation step, fixed numbers of purified B-cells were used (500 or 1,000 cells per well), and spots were counted using an ELISpot Reader (AID, Straßberg, Germany).

### Flow cytometry

**Figure 1:**
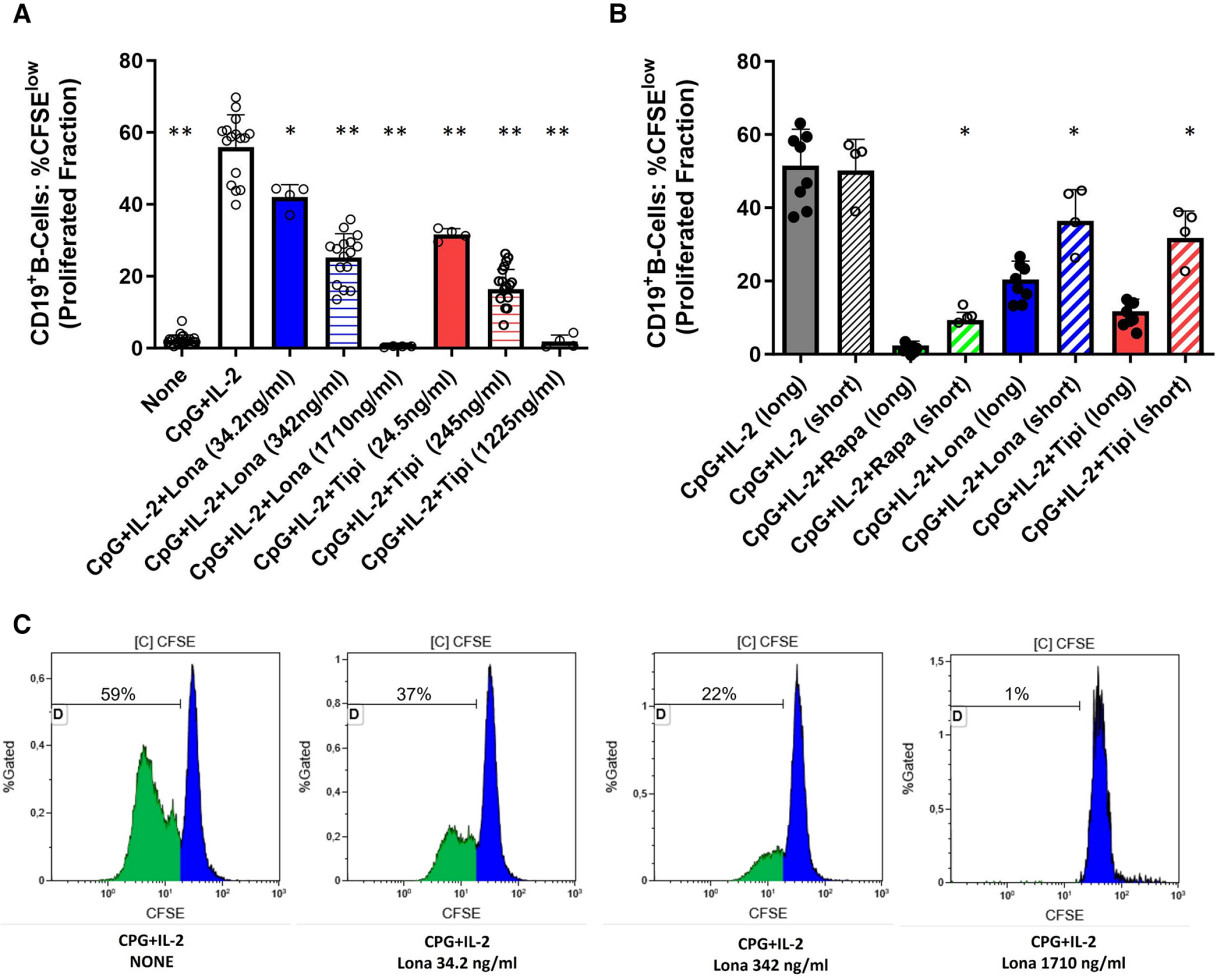
Dose-dependent suppression of TLR9-induced B-cell proliferation by FTI. (**A**) Purified B-cells from healthy donors were labeled with CFSE and stimulated with CpG plus IL-2 in the presence of different concentrations of FTI (tipifarnib and lonafarnib). After 72 h, CD19^+^ B-cell proliferation was determined by CFSE dilution. Stimulation with CPG + IL-2 in absence of FTI served as control condition. Statistical significance was calculated by repeated-measures ANOVA and corrections for multiple comparisons were done by Dunnett's test (all conditions were compared vs. CPG + IL-2; *p: < 0.05, **p: < 0.005). CpG + IL-2 *n* = 16, Lona (34.2 ng/ml) *n* = 4, Lona (342 ng/ml) *n* = 16, Lona (1710 ng/ml) *n* = 4, Tipi (24.5 ng/ml) *n* = 4, Tipi (245 ng/ml) *n* = 16, Tipi (1,225 ng/ml) *n* = 4. (**B**) B-cells from healthy donors were exposed either for only 24 h to FITs (short) or over the whole culture period (long). B-cells were stimulated in all conditions with CPG + IL-2. For short exposure to FTI, B-cells were washed after the first 24 h of culture followed by culture with CPG + IL-2 in the absence of FTI/rapamycin for an additional period of 48 h. Tipi and lona were used at concentrations of 245 ng/ml and 342 ng/ml, respectively. Statistical significance was calculated by repeated-measures ANOVA and corrections for multiple comparisons were done by Dunnett's test (the respective conditions with short exposure were compared to the matching conditions with long exposure. *p: < 0.05). CpG + IL-2 (long) *n* = 8, CpG + IL-2 (short) *n* = 4, Rapa (long) *n* = 8, Rapa (short) *n* = 4, Lona (long) *n* = 8, Lona (short) *n* = 4, Tipi (long) *n* = 8, Tipi (short) *n* = 4. (**C**) Representative flow cytometric data. Plots are gated on viable CD19**^+^** B-cells. Proliferation data was analyzed by gating on the cells that have undergone at least one round of division and is given as “Proliferated fraction”.

For GrB and IL-10 detection, purified B-cells were harvested at the end of the culture period and stained with an anti-CD19 Pacific Blue antibody (clone J3-119, Beckman Coulter, Krefeld, Germany) and 7AAD (BioLegend, Eching, Germany) followed by fixation/permeabilization (Cytofix/Cytoperm Kit, BD Biosciences, Heidelberg, Germany). B-cells were then intracellularly stained for GrB (anti-human GrB antibody, clone GB11, PE, eBioscience) or IL-10 (anti-human IL-10 antibody, APC, clone JES3-9D7, Biolegend). For the plasmablast formation assay, PBMC were harvested after culture and stained with anti-human CD19 (clone J3-119, FITC, Beckman Coulter), anti-human CD38 (clone HIT-2, PE, Biolegend) and anti-human CD27 (clone O323, APC-H7, Biolegend). Appropriate isotype controls were used to confirm the specificity of staining. Flow cytometric measurement was performed the same day by fluorescence activated cell sorting (FACS) with a NAVIOS^TM^ flow cytometer (Beckman Coulter). The software Kaluza, version 2.1 (Beckman Coulter) was used to analyze the FACS data. The gating strategy is depicted in Supplementary Figure 1. After gating on lymphocytes, viable B-cells were discriminated by 7AAD and CD19 staining. Viable cells were defined as CD19**^+^** B-cells being 7AAD**^neg^**. Subsequently, doublets were excluded. Proliferation data was analyzed by gating on the cells that have undergone at least one round of division and is given as “Proliferated fraction” (Figure 1). This parameter is most commonly used for the analysis of CFSE-derived data (14). The term “proliferated fraction” is synonymous with “Fraction diluted” and “Proliferative fraction”.

### Statistics

All values are expressed as the mean ± standard deviation (SD). Unless otherwise stated, statistical significance was calculated by repeated-measures ANOVA and corrections for multiple comparisons were done by Dunnett's test. A *p*-value below 0.05 was considered as statistically significant.

## Results

### FTI reduce CpG-induced B-cell proliferation

Purified B-cells from HC were used to investigate the effect of FTI on differentiation of B-cells. First, it was determined whether FTI have an impact on the proliferation of stimulated B-cells. For this purpose, CFSE-labeled B-cells were stimulated with CpG plus IL-2 in the presence or absence of FTI. Lonafarnib and tipifarnib suppressed B-cell proliferation in a dose-dependent manner (Figure 1). Viability was assessed after 72 h of culture and was assessed in two different ways: viable 7AAD**^neg^**CD19**^pos^** B-cells as percentage of total cells and viable 7AAD**^neg^** CD19**^pos^** B-cells within the CD19**^pos^** B-cell population. In absence of lonafarnib and tipifarnib, 69 ± 5% of total cells were CD19**^pos^**7AAD**^neg^** whereas 91 ± 3% within the CD19**^pos^** B-cell population were negative for 7AAD. At the lowest and intermediate concentrations of FTI, the overall fraction of CD19**^pos^**7AAD**^neg^** cells was slightly decreased as compared to stimulation without FTI. Viability within the B-cell population was comparable (total cells: %CD19**^pos^**7AAD**^neg^** and CD19**^pos^**B-cells: %7AAD**^neg^**; lona 34.2 ng/ml: 63 ± 4% and 91 ± 0.3%; tipi 24.5 ng/ml: 57 ± 2% and 89 ± 0.7%; lona 342 ng/ml: 50 ± 4% and 88 ± 1%; tipi 245 ng/ml: 51 ± 5% and 86 ± 2). At the highest concentration of FTI, the fraction of viable cells was greatly reduced (total cells: %CD19**^pos^**7AAD**^neg^** and CD19**^pos^** B-cells: %7AAD**^neg^**; lona 1710 ng/ml: 7 ± 2% and 41 ± 11%; tipi 1,225 ng/ml: 1.65 ± 0.2% and 9.21 ± 4%). Thus, high dosages of FTI induce cell death leading to decreased proliferation. Next, it was studied whether the suppressive effect of both agents is reversible. For this purpose, B-cells were exposed either for 72 h to FTI during stimulation with CPG and IL-2 or exposure to FTI during stimulation was limited to 24 h followed by FTI-free stimulation for another 48 h (Figure 1B). Suppression of B-cell proliferation was reduced during short term exposure vs. long term exposure. In a separate set of experiments, B-cells were exposed for 72 h to FTI while being stimulated followed by FTI-free stimulation for another period of 72 h. At the end of the second culture period, FTI-exposed B-cells showed reduced proliferation as compared to FTI-free conditions; however, proliferation of FTI-exposed B-cells was clearly enhanced when proliferation at the end of the second culture period was compared to the first culture period of 72 h (Supplementary Figure 2). Thus, the suppressive effects of FTI are potentially reversible.

### FTI diminish CpG-induced expression of the costimulator CD27 but not PDL-1

**Figure 2:**
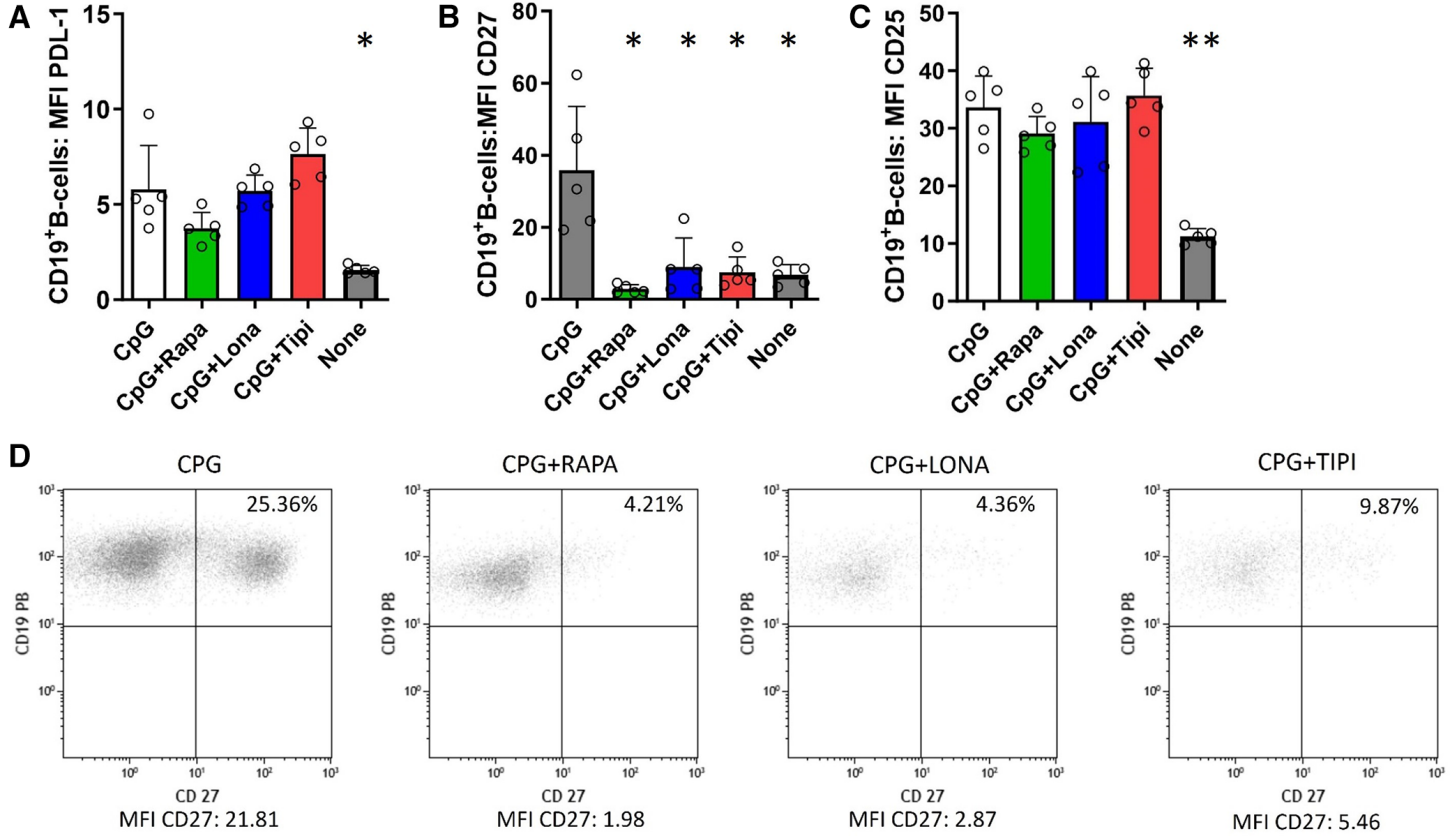
Effect of FTI on the expression of costimulatory molecules and CD25 on activated B-cells. CD19**^+^** B-cells from healthy donors were stimulated with CpG in the presence of FTI for 72 h. Rapamycin was used as a comparative immunosuppressive agent. Stimulation with CPG in absence of FTI served as control condition. Tipi and lona were used at concentrations of 245 ng/ml and 342 ng/ml, respectively.The expression of (**A**) PDL-1, (**B**) CD27 and (**C**) CD25 was analyzed by flow cytometry after 72 h of culture. For the determination of the MFI, cells were gated on viable CD19**^+^** B-cells (all conditions *n* = 5). Statistical significance was calculated by repeated-measures ANOVA and corrections for multiple comparisons were done by Dunnett's test (all conditions were compared vs. CPG; *p < 0.05, **p: < 0.005). (**D**) Representative flow cytometric data. Plots are gated on viable CD19**^+^** B-cells. B-cells with detectable expression of the respective costimulators or CD25 are depicted in the upper right quadrant.

To investigate whether FTI impact the costimulatory capacity of B-cells, the expression of PDL-1 and CD27 was studied. PDL-1 is a co-inhibitor, binds to PD-1 on T-cells and may mediate inhibitory signals to T-cells. CD27 serves on B-cells not only as a memory marker but also has a key role in promoting plasma cell differentiation as costimulator (15, 16). As expected, stimulation with CpG enhanced expression of PDL-1, CD27 and CD25. FTI treatment did not reduce the expression of PDL-1 on B-cells and was comparable to the control condition with CpG only; interestingly, rapamycin also had no influence on PDL-1 expression (Figure 2A). On the contrary, FTI and rapamycin diminished the fraction of CD27^+^ B-cells significantly (Figures 2B, 2D). There was no effect on CD25 expression (Figure 2C). However, the fraction of CD25 expressing B-cells was slightly reduced after treatment with Lonafarnib (Supplementary Figure 3).

### FTI interfere with the maturation of Il-10^pos^ and GrB^pos^ B-cells

**Figure 3:**
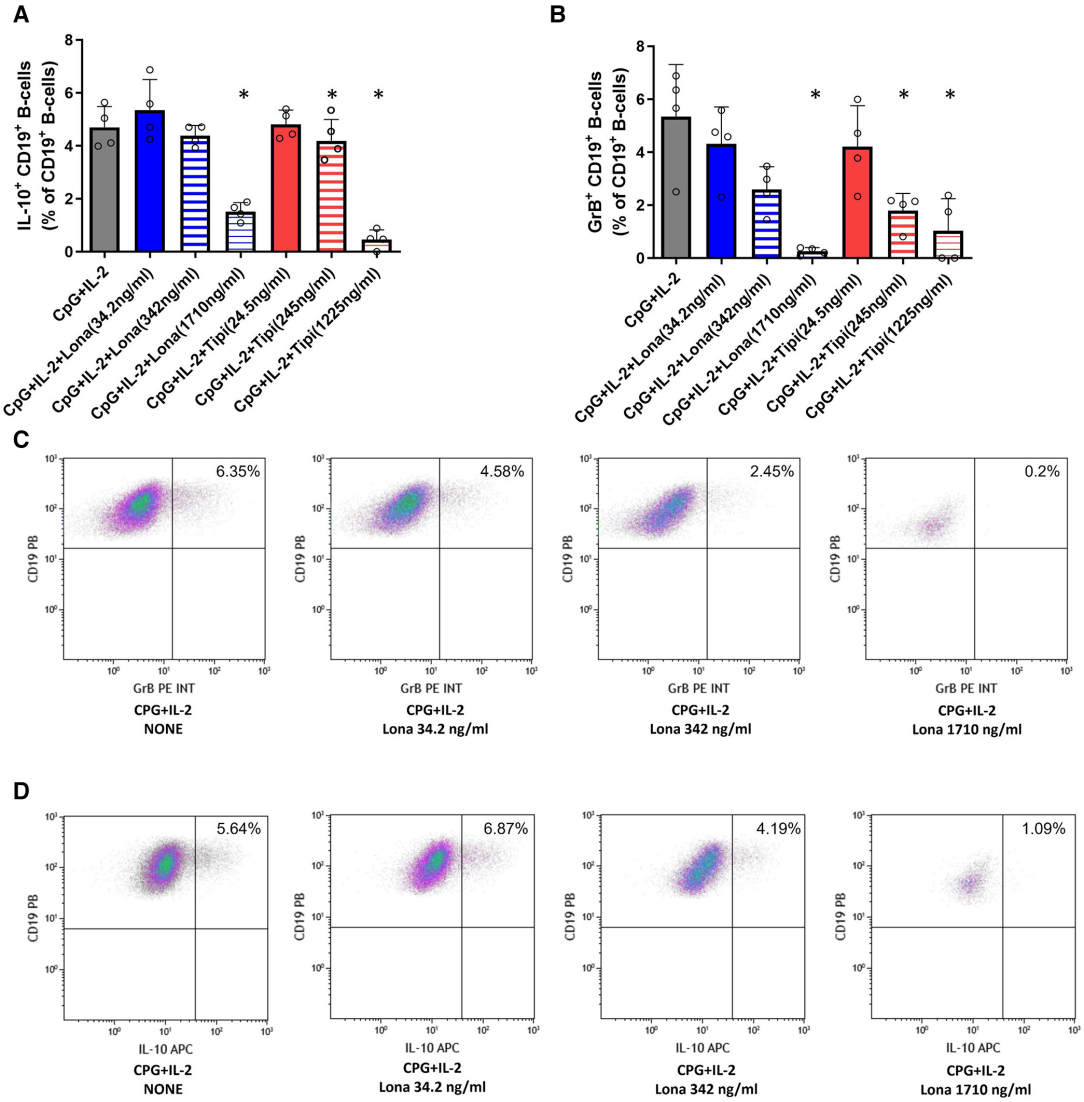
Impact of FTI on IL-10^+^ B-cells and GrB^+^ regulatory B-cells. B-cells from healthy donors were incubated for 72 h with FTI at different concentrations in the presence of CpG plus IL-2. After three days, the production of IL-10 and GrB by CD19^+^ B-cells was determined by flow cytometry. Stimulation with CPG + IL-2 in absence of FTI served as control condition. Tipi and lona were used at concentrations of 245 ng/ml and 342 ng/ml, respectively. (**A**) Impact of FTI on IL-10^+^ CD19^+^ B-cells upon CpG plus IL-2 stimulation (all conditions *n* = 4). (**B**) Impact of FTI on GrB^+^ CD19^+^ B-cells upon CpG plus IL-2 stimulation (all conditions *n* = 4). Statistical significance was calculated by repeated-measures ANOVA and corrections for multiple comparisons were done by Dunnett's test (all conditions were compared vs. CpG + IL-2; *p: < 0.05). (**C**) and (**D**) Representative flow cytometric data of GrB and IL-10 expressing B-cells. Plots are gated on viable CD19**^+^** B-cells. B-cells with detectable IL-10 or GrB expression are depicted in the upper right quadrant.

It was then tested whether FTI inhibit the maturation of IL-10 and GrB producing B-cells. For this purpose, IL-10 and GrB expressing B-cells were induced during cell culture. IL-10 as well as GrB production were assessed by flow cytometry. As expected, CpG + IL-2 was a potent inducer of IL-10**^pos^**and GrB**^pos^** B-cells (Figure 3). Lonafarnib lowered the fraction of IL-10**^pos^** B-cells at a higher concentration (1710 ng/ml) significantly whereas tipifarnib already diminished the fraction of IL-10^pos^ B-cells at a lower concentration (245 ng/ml) significantly (Figure 3). The same observation was made for GrB**^+^** B-cells (Figure 3). However, only at the highest concentrations, IL-10 and GrB production was almost completely suppressed with either tipifarnib or lonafarnib.

### FTI suppress plasmablast formation and IgG secretion in healthy controls and renal transplant patients

**Figure 4:**
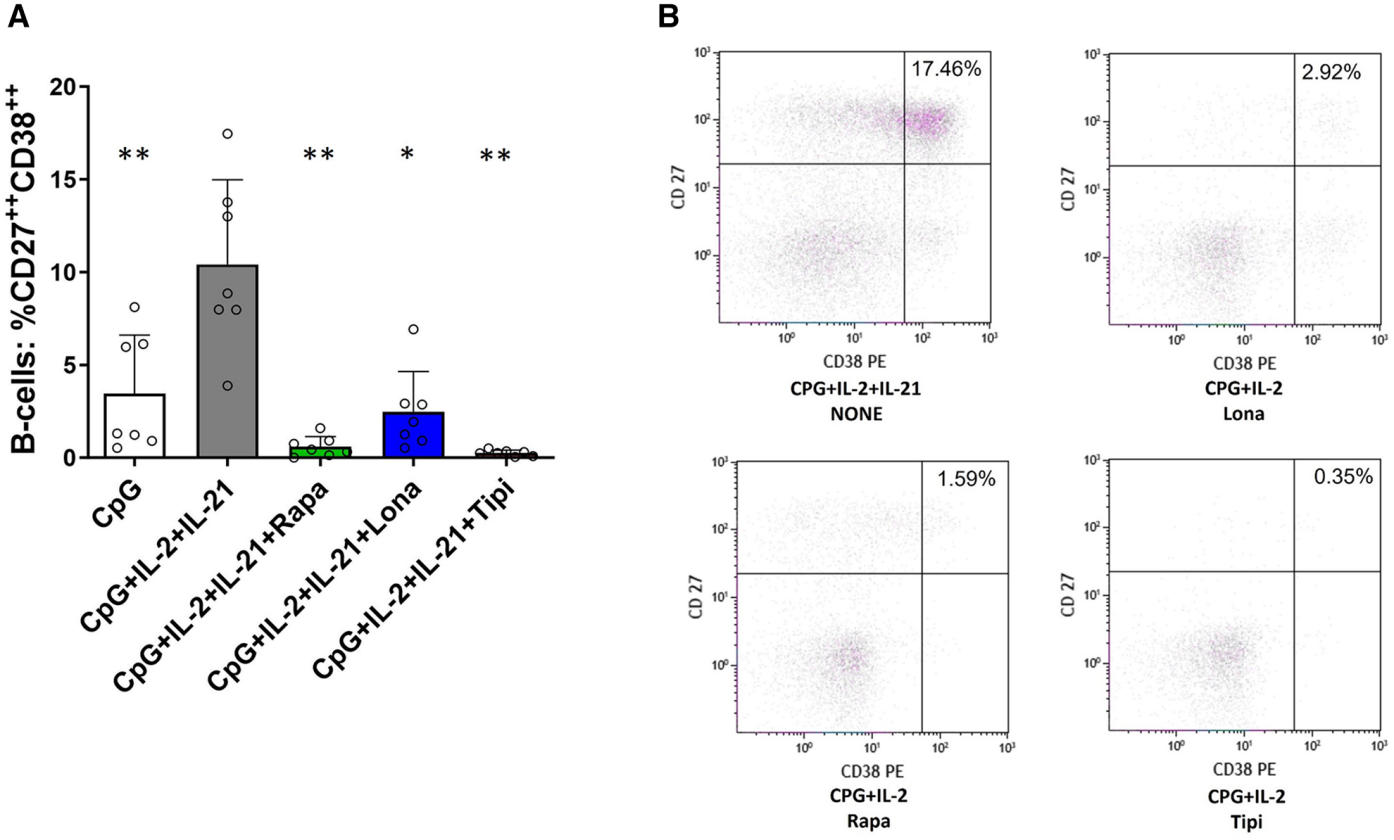
Impact of FTI on plasma cell formation. (**A**) Plasma cell formation is efficiently inhibited by FTI. B-cells from healthy donors were stimulated for 6 days in absence or presence of FTI. Differentiation into plasma cells was determined by flow cytometry (all conditions *n* = 7). Stimulation with CPG + IL-2 + IL-21 in absence of FTI served as control condition.Tipi and lona were used at concentrations of 245 ng/ml and 342 ng/ml, respectively. Statistical significance was calculated by repeated-measures ANOVA and corrections for multiple comparisons were done by Dunnett's test (all conditions were compared vs. CPG + IL-2 + IL-21, *p: < 0.05, **p: < 0.005). (**B**) Representative flow cytometric data. Plots are gated on viable CD19**^+^** B-cells. Plasma cells were defined as B-cells with high of expression of CD27 and CD38 (CD27**^++^**CD38**^++^**, upper right quadrant).

**Figure 5:**
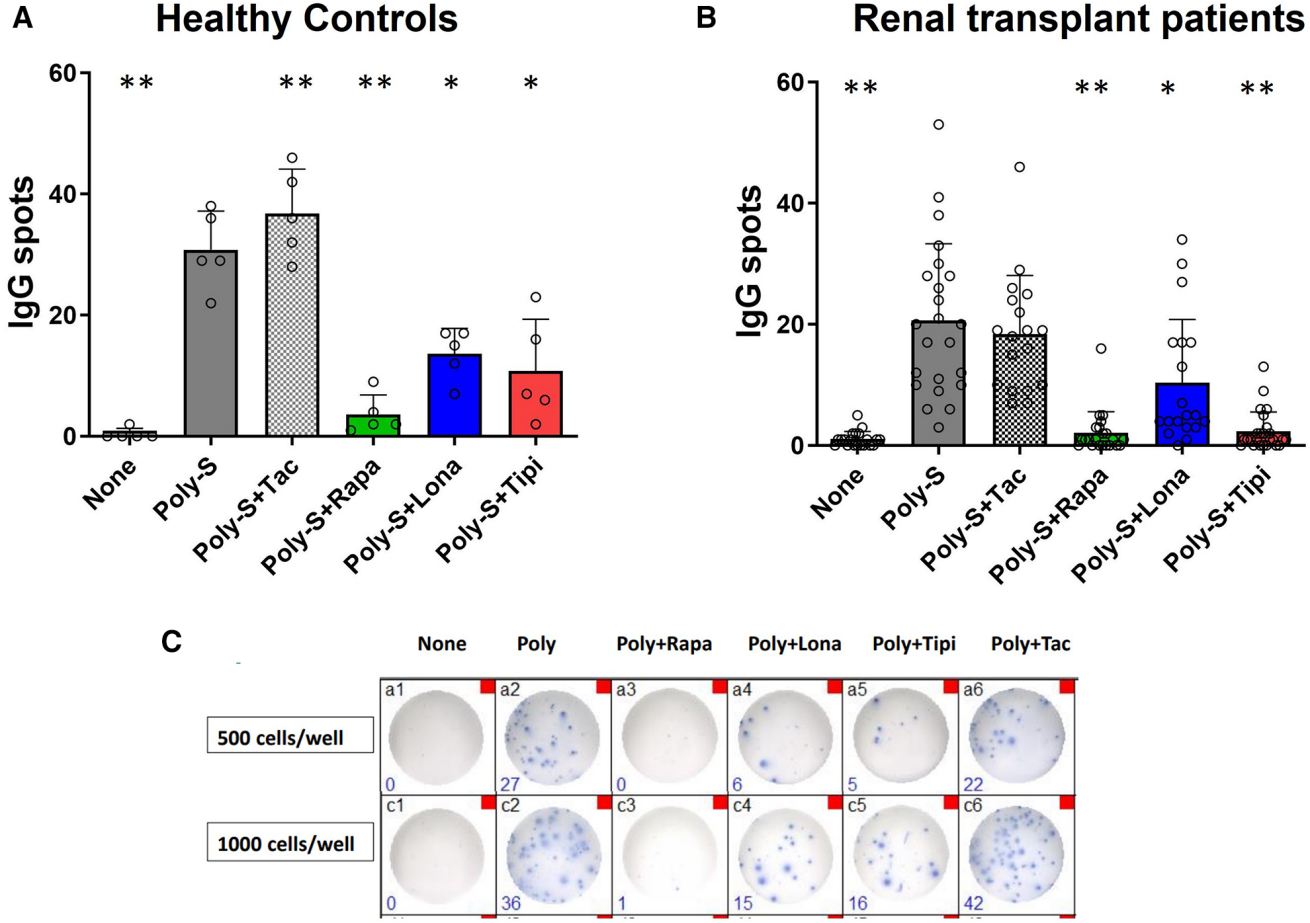
Effect of FTI on IgG secretion by plasma cells. Purified CD19^+^ B-cells were initially cultured at 5 × 10^4^ cells/well under Poly-S (10 μg/ml) stimulation in the presence of FTI for 4 days and then transferred to ELISpot plates at a density of 1,000 cells/well for another 24 h. Rapamycin and tacrolimus were used as controls. IgG secretion was detected by EliSpot. Tipifarnib (Tipi) and lonafarnib (Iona) were used at concentrations of 245 ng/ml and 342 ng/ml, respectively. (**A**) Impact of FTI on IgG secretion by plasma cells from HC (*n* = 5). (**B**) Impact of FTI on IgG secretion by plasma cells from renal transplant patients (*n* = 26; none *n* = 20, Poly-S *n* = 23, Poly-S + Rapa *n* = 22, Poly-S + Lona *n* = 19, Poly-S + Tipi *n* = 25, Poly-S + Tax *n* = 18). (**C**) Representative photos of IgG-specific ELISpot plates. Statistical significance was calculated by repeated-measures ANOVA and corrections for multiple comparisons were done by Dunnett's test (all conditions were compared vs. Poly-S, *p: < 0.05, **p: < 0.005).

Furthermore, the extent to which FTI impact effector B-cell function was investigated. Plasmablast formation and IgG secretion were studied. Plasmablast formation was induced by stimulation with CpG in the presence of IL-21 and IL-2 for six days. The fraction of plasmablasts was then determined by flow cytometry. Lonafarnib and tipifarnib efficiently inhibited plasmablast formation upon CpG stimulation being as potent as rapamycin (Figure 4). Subsequently, it was determined whether IgG secretion could be suppressed by FTI. Purified B-cells were stimulated for four days with Poly-S in the presence of the FTI, rapamycin or tacrolimus. Then, B-cells were transferred to ELISpot plates, and the number of spot-forming units (SFU) was determined after 24 h. Lonafarnib and tipifarnib significantly lowered the number of SFU and were equally potent to rapamycin (Figure 5A). Tacrolimus did not influence IgG secretion (Figure 5A). To assess whether FTI also show significant effects under disease conditions, B-cells derived from patients after renal transplantation were assayed. FTI inhibited IgG secretion to an extent similar to that seen in HC (Figures 5B,C).

## Discussion

In this study, it was shown that FTI efficiently suppress B-cell effector function *in vitro* and may interfere with maturation of B-cells which may possess regulatory activity. A very strong effect was observed on *in vitro* plasma cell formation and subsequent IgG secretion suggesting potent suppression of humoral immunity. B-cells from renal transplant patients were also susceptible *in vitro* to FTI-mediated suppression of humoral immunity.

FTI were originally developed as anti-cancer drugs targeting Farnesyltransferase (3, 5, 6). This enzyme drives the prenylation of the Ras protein. However, additional targets of Farnesyltransferase have been identified recently indicating that FTI may modulate additional relevant pathways apart from Ras (5, 6). Indeed, it was discovered that the disease-causing protein progerin in Hutchinson-Gilford progeria syndrome (HGPS) harbors a target site for farnesylation (17). Farnesylation of progerin causes accumulation of this aberrant protein. Clinical studies revealed that treatment with FTI reduced the accumulation of progerin and improved the prognosis of patients suffering from this disease. Lonafarnib was subsequently approved for treatment of patients with HGPS (17). Thus, FTI also have clinical relevance in other medical contexts than originally anticipated (5).

Additional studies suggest that FTI have immunomodulatory activity. In an animal model for rheumatoid arthritis (RA), treatment with FTI decreased synovial TNF and IL-1 mRNA expression dampening inflammation (18). Similarly, tipifarnib largely prevented the onset of concavalin-A induced liver disease in a murine model for autoimmune hepatitis (19). The authors suggested that the modulation of pro-inflammatory T-cell immunity mediated protection from disease in this model. Likewise, *in vivo* treatment of mice after allogeneic skin transplantation with FTI delayed the onset of rejection (11). The authors showed that the allogeneic T-cell response was suppressed *in vitro* by FTI and that the production of pro-inflammatory T-cell derived cytokines was inhibited. Puniya et al. recently predicted in a computational model based on multi-omic data Farnesyl-diphosphate farnesyltransferase 1 (FDFT1) as drug target candidate in T-cell mediated diseases such as RA and multiple sclerosis (20). FDFT1 is a key enzyme in the mevalonate metabolism of T-cells and the corresponding metabolites of this pathway regulate T-cell activation (21). Thus, FTI may offer the opportunity for targeted treatment of immune cells. There are few studies that assessed the impact of FTI on B-cells. In a murine model for B-cell lymphoma, FTI prolonged survival by blocking proliferation of transformed B-cells (12). In another study by Marzo et al., leukemic B-cells isolated from patients were susceptible to FTI-induced apoptosis (22). We found that lonafarnib and tipifarnib both inhibited TLR-9 induced proliferation of B-cells which was dose-dependent. Shimabukuro-Vornhagen et al. studied the metabolic conditions of CD40-dependent B-cell activation (23). They found that depletion of mevalonate with statins led to suppression of B-cell proliferation. However, suppression was not observed when an FTI was used for this purpose. Furthermore, the authors demonstrated that TLR9-induced B-cell activation was not suppressed by statins suggesting that mevalonate depletion is not relevant to all types of B-cell activation. Our study extends the findings of Shimabukuro-Vornhagen and provides further evidence that FTI suppress specific types of B-cell activation, i.e., TLR9-induced B-cell activation. Furthermore, we did not observe overall suppression of B-cell activity by FTI. With respect to IL-10/GrB production and humoral effector functions of B-cells, a differential impact was observed. IL-10 and GrB producing B-cells were studied. Both types have been shown to play a key role in maintaining immune tolerance in autoimmunity and renal transplantation in the past (24–27). However, recent data indicated that IL-10 producing B-cells dot not necessarily possess regulatory, anti-inflammatory activity (28). IL-10 producing B-cells seemed less susceptible to FTI-mediated inhibition than GrB producing B-cells. Only at very high concentrations of FTI, maturation of IL-10 producing B-cells was suppressed. Bibby et al. studied the interplay between cholesterol metabolism and IL-10**^+^** B-cell development (29). Although the stimulation protocol was different from ours, the FTI used by Bibby et al. did not impact B-cell development. Another regulatory mechanism commonly used by B-cells with anti-inflammatory activity is co-inhibition (30, 31). Co-inhibition is mediated by antigen-presenting cell like B-cells and interferes with T-cell activation via e.g., PD-1/PDL-1 interaction. Therefore, the expression of PDL-1 on B-cells was investigated and did not significantly change under *in vitro* treatment with FTI. Overall, potential regulatory mechanisms of B-cells were largely preserved despite *in vitro* treatment with FTI. Effector functions of B-cells were studied, too. Plasma cell formation was very susceptible to FTI treatment and was almost abolished with tipifarnib. Accordingly, IgG secretion was sharply reduced in presence of FTI. Thus, humoral immunity was susceptible to FTI treatment while other functions of B-cells were preserved. In the context of renal transplantation, humoral rejection is a serious problem which limits the renal allograft survival and efficacious treatment strategies are lacking (2). Given the findings in healthy controls, we sought to study the *in vitro* activity of FTI on humoral effector B-cell function in renal transplant patients. Interestingly, both FTI tested showed significant suppression of plasma cell formation and IgG secretion. Therefore, also in patients with persistent activation of humoral immunity, FTI had a potent immunomodulatory impact on effector B-cells *in vitro*. However, the heterogeneity of the patient population with regard to immunosuppressive treatment is a limitation of this study. Further *in vitro* studies are needed to decipher and characterize the immunomodulatory potency of FTI in solid organ transplantation.

In summary, we demonstrated that FTI suppress CpG-induced effector B-cell maturation and potently inhibit humoral immunity. Furthermore, this effect was not limited to B-cells from healthy controls but was also reproducible in B-cells from patients after renal transplantation. In addition, other types of B-cell function such as production of IL-10 and GrB with potential regulatory importance were preserved *in vitro*. These novel insights may encourage further studies to investigate the value of FTI as immunomodulatory agent.

## Data Availability

The raw data supporting the conclusions of this article will be made available by the authors, without undue reservation.
