# Supplementary material for: Farnesyltransferase-inhibitors exert in vitro immunosuppressive capacity by inhibiting human B-cells
Source: Front Transplant. 2023 Nov 9;2:1233322. doi: 10.3389/frtra.2023.1233322 (PMC11235315; doi:10.3389/frtra.2023.1233322)
Supplement: Supplementary file 1 [file Datasheet1.pdf]

Supplementary Data

Manuscript ID 1233322: "Farnesyltransferase-inhibitors exert immunosuppressive capacity by inhibiting human B-cells"

Gating Strategy

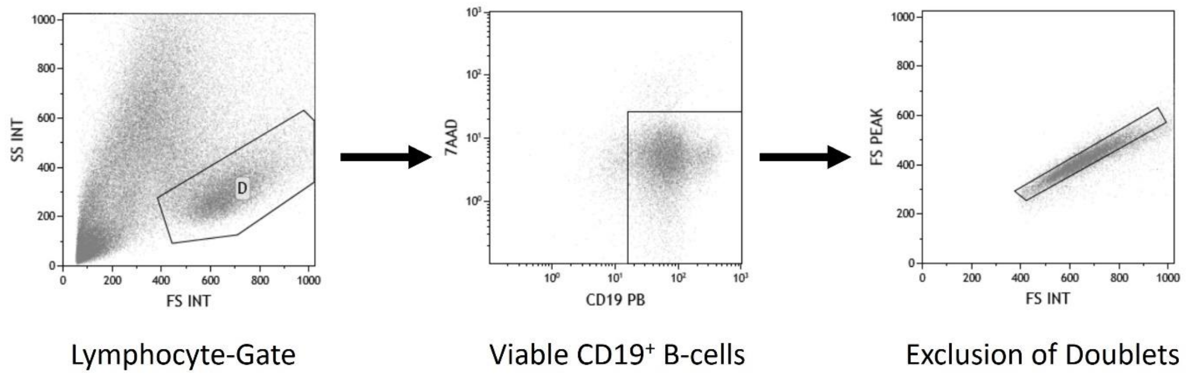

Supplementary Figure 1. Gating strategy.
Either PBMC or purified B-cells were the source for analysis. After gating on lymphocytes, viable B-cells were discriminated by 7AAD and CD19 staining. Viable cells were defined as CD19^{pos} B-cells being 7AAD^{neg}. Subsequently, doublets were excluded.

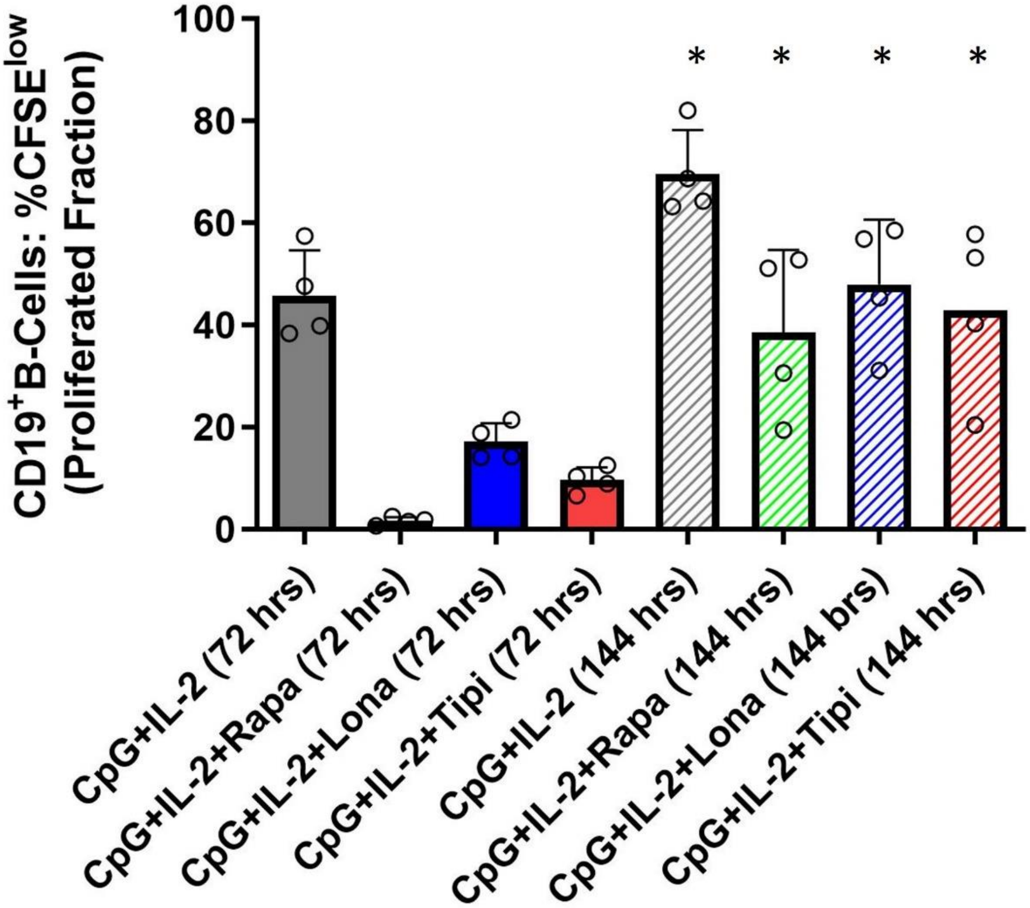

Supplementary Figure 2. Exposure time to FTI and impact on B-cell proliferation. B-cells were exposed for 72 hours to FTI while being stimulated with CPG and IL-2. As control condition, B-cells were stimulated with CPG and IL-2 in absence of FTI (n=4 for all conditions). After 72 hours, in all conditions cells were washed followed by FTI-free stimulation with CPG and IL-2 for another period of 72 hours. In addition, replicates were measured already after the first 72 hours of incubation to determine the proliferated fraction. At the end of the second culture period (144 hrs), FTI-exposed B-cells showed reduced proliferation as compared to FTI-free conditions (i.e. CPG+IL-2). However, proliferation of FTI-exposed B-cells was clearly enhanced when proliferation at the end of the second culture period (144 hrs) was compared to matching conditions of the first culture period with 72 hrs. Tipi and lona were used at concentrations of 245 ng/ml and 342 ng/ml, respectively. Statistical significance was calculated by repeated-measures ANOVA and corrections for multiple comparisons were done by Dunnett's test (the respective conditions with 144 hrs of culture were compared to the matching conditions with 72 hours of culture, *p: <0.05).

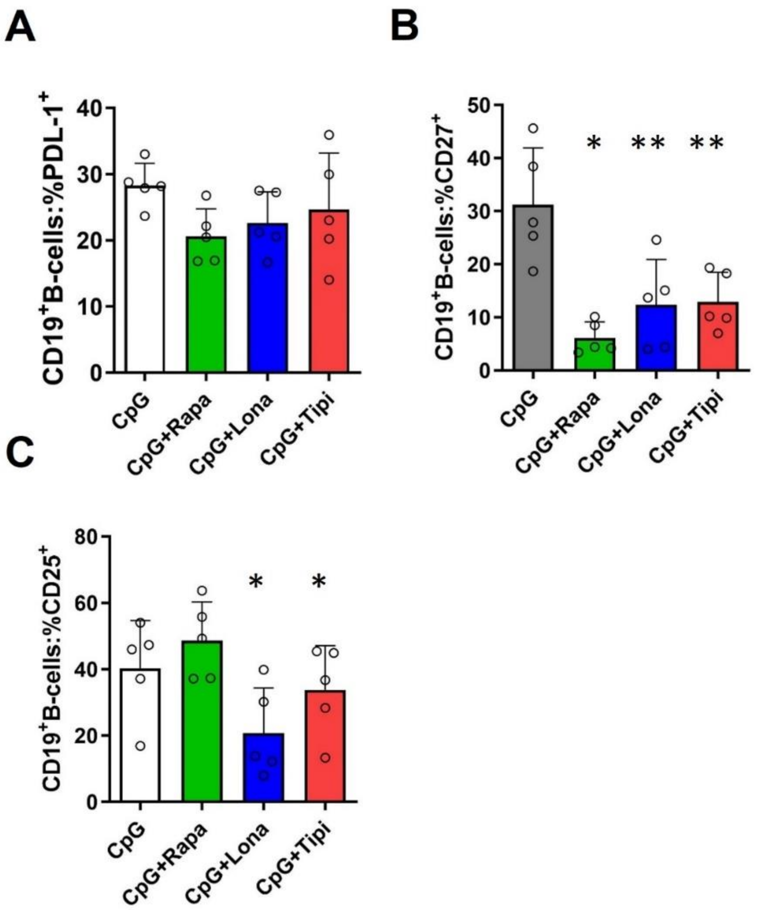

Supplementary Figure 3. Effect of FTI on the expression of costimulatory molecules and CD25 on activated B-cells.

CD19pos B-cells from healthy donors were stimulated with CpG in the presence of FTI for 72 hours. Rapamycin was used as a comparative immunosuppressive agent. Stimulation with CPG in absence of FTI served as control condition control. Tipi and lona were used at concentrations of 245 ng/ml and 342 ng/ml, respectively. The fraction of B-cells expressing (A) PDL-1, (B) CD27 and (C) CD25 was analyzed by flow cytometry after 72 hours of culture (all conditions n=5). Statistical significance was

calculated by repeated-measures ANOVA and corrections for multiple comparisons were done by Dunnett's test (all conditions were compared vs. CPG; *p<0.05, **p: <0.005).
